# Temperature and population density: interactional effects of environmental factors on phenotypic plasticity, immune defenses, and disease resistance in an insect pest

**DOI:** 10.1002/ece3.2158

**Published:** 2016-04-27

**Authors:** Farley W. S. Silva, Simon L. Elliot

**Affiliations:** ^1^ Post‐graduate Program in Entomology Department of Entomology Universidade Federal de Viçosa (UFV) Av. PH Rolfs 36570‐900 Viçosa Minas Gerais Brazil

**Keywords:** AgMNPV, climate change, density‐dependent prophylaxis, immune responses, phenotypic plasticity, velvetbean caterpillar (*Anticarsia gemmatalis*)

## Abstract

Temperature and crowding are key environmental factors mediating the transmission and epizooty of infectious disease in ectotherm animals. The host physiology may be altered in a temperature‐dependent manner and thus affects the pathogen development and course of diseases within an individual and host population, or the transmission rates (or infectivity) of pathogens shift linearly with the host population density. To our understanding, the knowledge of interactive and synergistic effects of temperature and population density on the host–pathogen system is limited. Here, we tested the interactional effects of these environmental factors on phenotypic plasticity, immune defenses, and disease resistance in the velvetbean caterpillar *Anticarsia gemmatalis*. Upon egg hatching, caterpillars were reared in thermostat‐controlled chambers in a 2 × 4 factorial design: density (1 or 8 caterpillars/pot) and temperature (20, 24, 28, or 32°C). Of the immune defenses assessed, encapsulation response was directly affected by none of the environmental factors; capsule melanization increased with temperature in both lone‐ and group‐reared caterpillars, although the lone‐reared ones presented the most evident response, and hemocyte numbers decreased with temperature regardless of the population density. Temperature, but not population density, affected considerably the time from inoculation to death of velvetbean caterpillar. Thus, velvetbean caterpillars succumbed to *Anticarsia gemmatalis multiple nucleopolyhedrovirus* (AgMNPV) more quickly at higher temperatures than at lower temperatures. As hypothesized, temperature likely affected caterpillars' movement rates, and thus the contact between conspecifics, which in turn affected the phenotypic expression of group‐reared caterpillars. Our results suggest that environmental factors, mainly temperature, strongly affect both the course of disease in velvetbean caterpillar population and its defenses against pathogens. As a soybean pest, velvetbean caterpillar may increase its damage on soybean fields under a scenario of global warming as caterpillars may reach the developmental resistance faster, and thus decrease their susceptibility to biological control by AgMNPV.

## Introduction

Temperature and crowding are key environmental factors that mediate the transmission and epizootiology of infectious diseases in ectothermic animals. The host's physiology can be altered in a temperature‐dependent manner and thus indirectly affects the course of disease both within an individual and in a host population (Johnson et al. 1982; Paaijmans et al. 2010). Meanwhile, transmission rates (or infectivity) of pathogens shift linearly with host population density (Anderson and May 1981). While the knowledge of the separate effects of environmental factors on immunological and resistance responses of hosts against pathogens is broad, our understanding the interactive and synergistic effects of such environmental factors on the host–pathogen system is limited.

A range of behavioral and physiological processes of animals are temperature‐dependent, being usually, but not always, maximized in ectotherm organisms experiencing warmer temperatures (Browning 1952; Lafrance 1968; Okasha 1968; Schramm 1972; Inglis et al. 1996). Furthermore, temperature may play a critical role, not just in the behavioral and physiological processes of the organism *per se*, but also in the host–pathogen interactions (Murdock et al. 2012b). For instance, thermoregulation and expression of behavioral fever in *Schistocerca gregaria* dramatically reduce its chance of succumbing to fungal disease (Elliot et al. 2002).

However, warmer temperatures may also be stressful to ectotherm organisms. This may negatively impact other biological parameters, such as reproduction in the diamondback moth *Plutella xylostella* (Zhang et al. 2013), or interactions with bacterial endosymbiont in ants (Fan and Wernegreen 2013). Changes in environmental temperature can also affect immune systems, hence affecting the animal's capacity to defend itself against pathogens. Adult white shrimp *Litopenaeus vannamei*, for example, had some of its immunological parameters negatively affected after exposure to thermal stress (Diaz et al. 2013). In insects, the effects of temperature on the components of immune system are quite varied (Karl et al. 2011; Catalan et al. 2012; Murdock et al. 2012a), likely due to species‐specific variability and the spectrum of immune parameters assessed in the studies.

While changes in the thermal environment may trigger direct physiological and behavioral responses at the organism level, it may also indirectly trigger such responses through alternative pathways. In insects, for example, a greater resistance to a pathogenic challenge can be reached via changes in cuticular melanization, which enhances the immune investment pleiotropically (Fedorka et al. 2013; Kutch et al. 2014). Moreover, environmental cooling or warming may affect the likelihood of transmission or impact of disease at both organism and population levels. In a lepidopteran virus system, disease transmission and outbreak intensity increase at warmer temperatures via consumption of contaminated food (Elderd and Reilly 2014). The mobility (and probably contact between conspecifics) of insects also increases with temperature (Ruf and Fiedler 2002; Cormont et al. 2011), so it might be expected that the risk of becoming infected by horizontally‐transmitted pathogens also increases (i.e., it is more likely to find contaminated food or infected conspecifics).

Nonetheless, some organisms have evolved prophylactic responses to cope with the increased risk of infection under such circumstances (density‐dependent prophylaxis hypothesis, Wilson and Reeson 1998). The DDP hypothesis predicts that (1) the individual mortality rate, upon a parasitic challenge, will decrease with prior experiences of increase in host density; (2) the individual investment in resistance will be proportional to the increase in host density; and (3) the host mortality induced by parasites in the field will saturate with host density (Wilson and Cotter 2009). This phenomenon has been broadly supported with studies on secondary defenses of animals (e.g., insects, sea stars, and birds) subject to variable population densities (Møller et al. 2003; Cotter et al. 2004; Bailey et al. 2008; Mills 2012).

**Figure 1:**
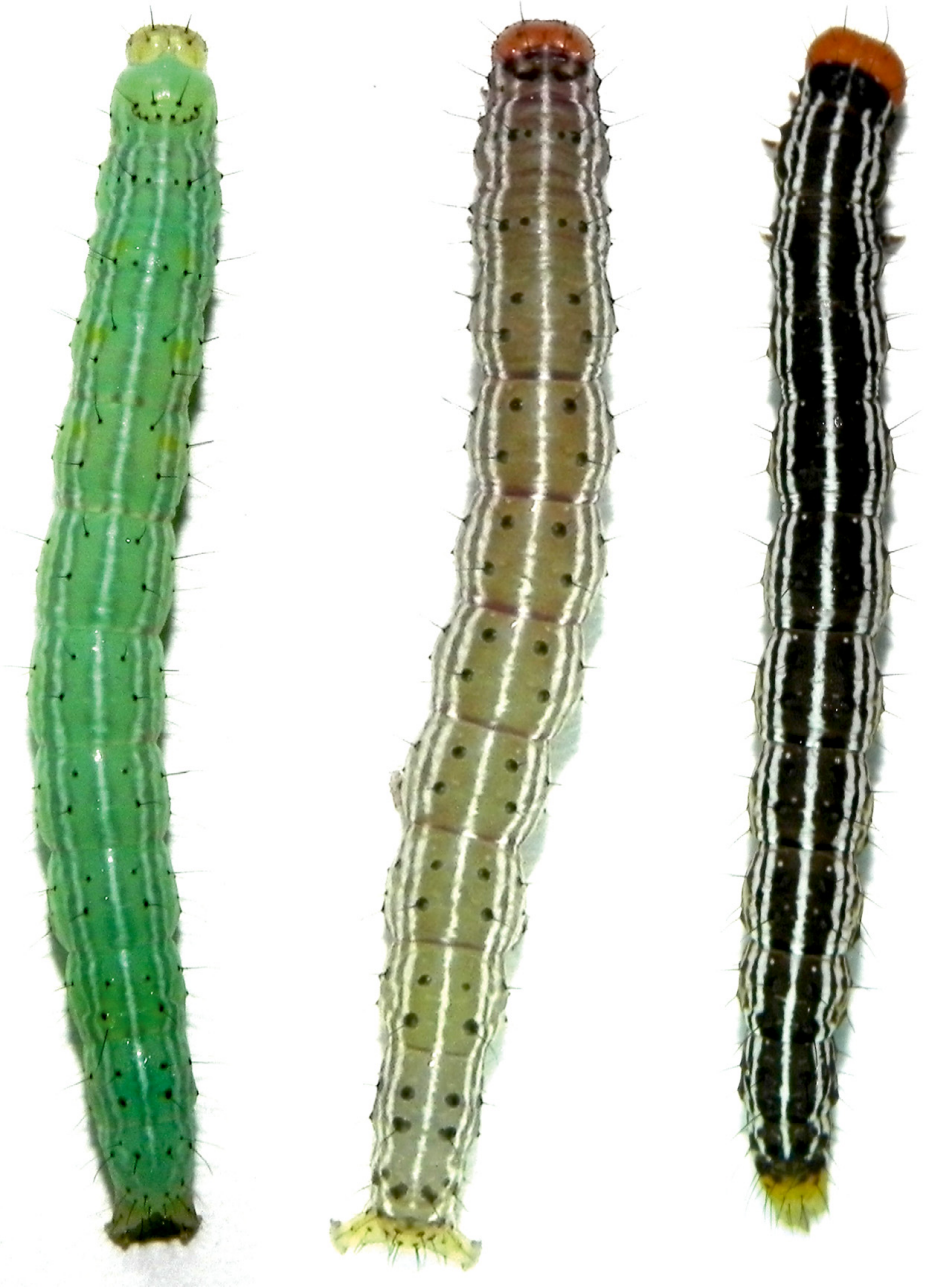
*Anticarsia gemmatalis* larvae “expressing” phenotypes: green, intermediate, or black.

It was shown recently that the velvetbean caterpillar *Anticarsia gemmatalis* (Fig. 1), when exposed to a high population density, beyond undergoing changes in its secondary defenses (i.e., encapsulation response, capsule melanization, and hemocyte numbers (Silva et al. 2013), also change plastically its primary defenses, such as thickness of the midgut epithelium and thickness and chitin amount in the peritrophic matrix (Silva et al. 2016). As other insect polyphenic species (e.g., Reeson et al. 1998; Barnes and Siva‐Jothy 2000; Wilson et al. 2001), the velvetbean caterpillar becomes darker (or melanized) as a response to the local density of conspecifics, with this feature being indicative of up‐regulation of primary and secondary defenses, and consequently, pathogen resistance. The velvetbean caterpillar is one of the main pests of soybean (*Glycine max*), and insecticides are commonly used to control it. However, this control strategy has several adverse environmental impacts and can lead to insecticide resistance (Abot et al. 1996). Hence, biological control [mainly by *Anticarsia gemmatalis multiple nucleopolyhedrovirus* (AgMNPV)] arises as an important component of soybean‐integrated pest management (IPM). This species‐specific virus occurs naturally in velvetbean caterpillar populations, presenting thus a potential control agent in soybean IPM programs (Moscardi 1989, 1999).

**Figure 2:**
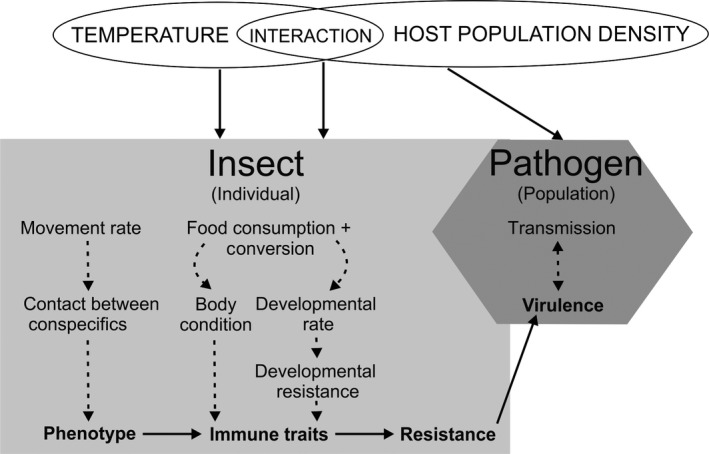
Schematic framework describing the direct (solid arrows) and indirect (dashed arrows) effects of environmental factors (inside the ellipses) on the insect–pathogen system (parameters assessed here are in bold). Temperature may affect caterpillars' movement rates, and hence the contact between conspecifics, which in turn affects the phenotypic expression of group‐reared caterpillars (Fig. 1). This species (as other phase polyphenic species) uses population density as cues for the risk of pathogen transmission; thus, it regulates plastically immune defenses (or phenotype) in response (see Silva et al. 2013). Temperature may also affect immune defenses, and consequently the insect resistance to pathogens, by changing the caterpillar's body condition and developmental rate (which presumably affects the “developmental resistance”; see Discussion).

We hypothesize here that environmental factors such as temperature and population density affect either directly or indirectly the immune defenses in the velvetbean caterpillar, and hence its capacity to survive to pathogens attack (see schematic framework in Fig. 2). Temperature may indirectly affect the phenotypic expression of group‐reared caterpillars by changing caterpillars' movement rates, and thus the contact between conspecifics. *Anticarsia gemmatalis* uses contact as cues for the increased risk of pathogen transmission at high population density; thus, it regulates plastically phenotypic traits (such as immune defenses) in response (see Silva et al. 2013). Temperature may also indirectly affect immune defenses, and consequently the insect resistance to pathogens, by changing the caterpillar's body condition and developmental rate [which presumably affects the “developmental resistance”; that is, larvae become more resistant to baculovirus infection as they age (Engelhard and Volkman 1995)].

## Materials and Methods

### The velvetbean caterpillar *Anticarsia gemmatalis*

The colony was first established in 2009 at the Laboratory of Insect–Microbe Interactions at the Universidade Federal de Viçosa (UFV), Brazil, from a stock colony maintained at EMBRAPA/CNPSo. Moths were housed in groups of *ca*. 40 pairs in wooden cages (measuring 30 × 30 × 30 cm) and fed *ad libitum* with artificial diet (10.5 g honey, 1.05 L water, 350 mL beer, 60 g sucrose, 1.05 g ascorbic acid, and 1.05 g methyl parahydroxybenzoate). They were allowed to oviposit on sheets of sulfite paper at 27°C and 14 h photophase. Eggs were collected daily and kept in plastic pots (500 mL) containing artificial diet for newly hatched caterpillars [as described by Hoffmann‐Campo et al. (1985)]. Upon egg hatching, caterpillars were promptly placed in opaque plastic pots (100 mL) and assigned to a 2 × 4 factorial design with two levels of density [1 or 8 caterpillars/pot; see (Silva et al. 2013)] and four levels of temperature (20, 24, 28 or 32°C). Temperatures were obtained using thermostat‐controlled chambers that were kept in a room with controlled conditions: 20 ± 1°C, 60 ± 3% relative humidity and 12 h photophase. To avoid pseudoreplication, only one caterpillar, randomly selected, from a given pot was ever used in a given treatment.

### Color phenotypic changes in caterpillars reared at different temperatures and population densities

**Figure 3:**
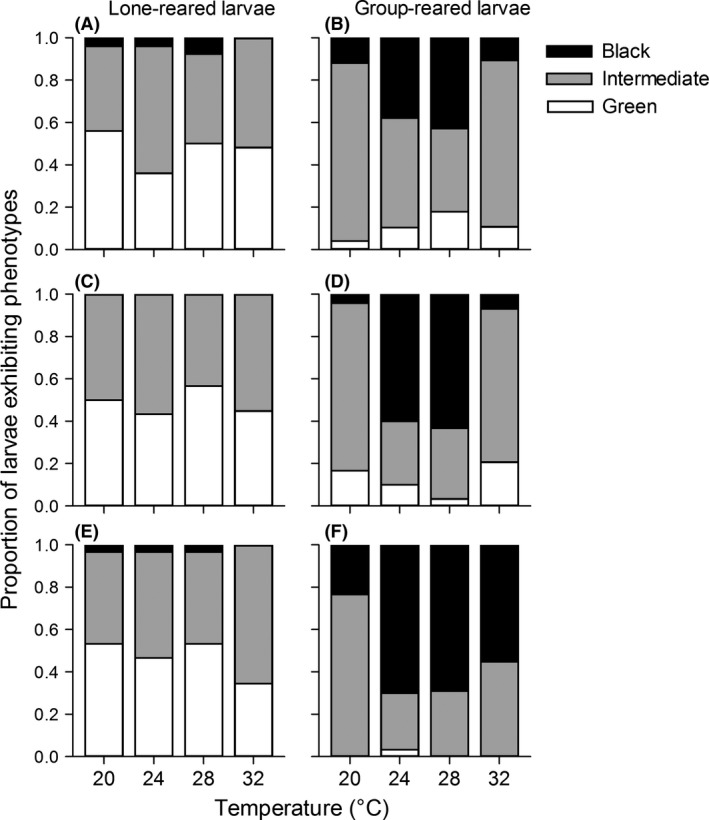
Frequency distribution of velvetbean caterpillars (*Anticarsia gemmatalis*) exhibiting color phenotypes according to two environmental factors, temperature and population density. Caterpillars were kept alone or in groups of 8 in four thermostat‐controlled chambers with a different temperatures (20, 24, 28, or 32°C) and exhibited the phenotypes green, intermediate, or black as a result (see statistical tests in text). Frequency distributions are shown for insects used subsequently in each experiment: (A,B) Experiment 1, encapsulation response and capsule melanization, (C,D) Experiment 2, hemocyte numbers, and (E,F) Experiment 3, susceptibility to AgMNPV.

The velvetbean caterpillar exhibits different color phenotypes—green, intermediate, or black—when in the presence or absence of conspecifics (see Fig. 1). Part of this phenotypic change is under genetic control, so “green phenotypes” may be found at a high density and “black phenotypes” at a low density; although in our study system the frequency of these phenotypes under such circumstances is very rare (see Fig. 3). Thus, most caterpillars at low densities express the green phenotypes, whereas caterpillars at high densities express the black phenotypes. The phenotypes were determined in all of the following experiments, taking into account the head capsule and body colorations [see Fig. 1 and Silva et al. (2013)].

### Experiment 1: Effects of temperature and population density on the encapsulation and melanization responses in caterpillars

Our aim here was to examine the effects of temperature and population density on immune defenses (i.e., encapsulation response, capsule melanization, and hemocyte numbers) and in particular to determine whether these factors interacted in their effects. These parameters were chosen because of their simplicity to be measured, and also because of their effectiveness against viral infections in closely related systems, that is, baculovirus‐lepidopteran pests. In brief, hemocytes are responsible for encapsulation of infected cells (or infection foci), while other biochemical pathways for capsule melanization, which together clear virus from the hemolymph (Washburn et al. 1996; Trudeau et al. 2001). As temperature may indirectly affect caterpillar immune defenses by effects on body mass, we weighed our caterpillars on scales (accuracy = 0.001 g) before each experiment. Temperature may also affect caterpillars' movement rates (see Fig. 2) but we did not assess this factor in our experiment as we felt that to do so might interfere in the physiological responses. Two immune functions were assessed in this experiment: encapsulation response and capsule melanization. They were measured by challenging fourth‐instar caterpillars, reared in the density and temperature treatments as above, with a colorless nylon filament (2 mm length and 0.12 mm Ø) that mimics the presence of a parasite, and thus the host immune response toward invasion (Schmid‐Hempel and Schmid‐Hempel 1998). The nylon filament was inserted through the first thoracic segment (dorsal region) of 30 caterpillars per treatment. Twenty‐four hours after this, caterpillars were dissected and the nylon filaments were mounted on slides and photographed. We could thus assess the area of cell layers formed around the nylon filament (encapsulation response) and the grayscale range (capsule melanization) with the aid of IMAGEJ 1.42q software (National Institutes of Health, Bethesda, MD, USA; http://imagej.nih.gov/ij/).

### Experiment 2: Effects of temperature and population density on hemocyte numbers

Again, the aim was to examine effects of temperature and population density (including interactions between the two factors) on investment in an immune defense, in this case, hemocytes. Caterpillars were reared in the density and temperature treatments (as above) until the fourth instar. In this stage, 30 caterpillars per treatment were used; a small hole was made with a sterile entomological pin between the first and second proleg and hemolymph was collected. A sample of 5 *μ*L of hemolymph was collected and added to an Eppendorf tube with 20 *μ*L anticoagulant buffer (98 mmol/L NaOH, 186 mmol/L NaCl, 17 mmol/L Na2 EDTA, and 41 mmol/L Citric acid, pH 4.5) plus 12 *μ*L of Giemsa stain [adapted from Ibrahim and Kim (2006)]. Two aliquots of 8 *μ*L of the suspension were added in each side of a Neubauer improved chamber and total hemocytes were counted under a microscope. The final value was the mean of the two aliquots, providing the cell numbers per microliter.

### Experiment 3: Effects of temperature and population density on infection by *Anticarsia gemmatalis multiple nucleopolyhedrovirus* (AgMNPV)

Here, we sought to determine how temperature and population density (and their interaction) might affect resistance to an important pathogen. Thirty, fourth‐instar caterpillars per treatment (as above) were kept in isolation and starved for 24 h before inoculation with AgMNPV. They were infected by feeding for a 24‐h period on square soybean leaf pieces (15 × 15 mm) inoculated with 20 *μ*L of virus suspension (6 × 10^6^ polyhedra/caterpillar). This viral concentration was chosen based on previous work (Silva et al. 2013), in which a discriminatory dose, in preliminary tests, showed that virus started to kill larvae around the fifth day after inoculation. The leaf piece is easily consumed by one caterpillar in a single day (ensuring ingestion of a uniform number of viral particles leading to infection); caterpillars that did not consume the entire leaf piece in this period were excluded from the experiment. Virus‐inoculated caterpillars were kept as above, and mortality was assessed daily until death or pupation.

### Data analysis

Pearson's chi‐squared test was applied on contingency tables to test the hypothesis of independence of the frequency distribution of caterpillars exhibiting phenotypes under different temperatures and population density.

For Experiments 1 and 2, we tested the effects of temperature and population density (as main factors), and phenotype and weight (used as covariates) on immunity parameters of velvetbean caterpillar. We first fitted full models using GLM (generalized linear models) with normal distributions. Subsequent simplification was made by excluding nonsignificant terms. Final models were accepted when not significantly different from the previous models. Residuals of the final model were checked for suitability of the distribution.

Survival data (Experiment 3) were analyzed by GLM with Weibull distribution; censoring data were used when caterpillars pupated. These models were performed including temperature, population density, and caterpillars' phenotypes as factors (Crawley 2007). Finally, contrast analysis was carried out to check the differences among factor levels of the two variables, temperature and phenotype.

Throughout the text, means ± standard errors (SE) are presented. All analyses were conducted in R version 3.0.3.

## Results

### Color phenotypic changes in caterpillars reared at different temperatures and population densities

The frequency distribution of color phenotypes in velvetbean caterpillars is dependent on both temperature and population density (Fig. 3). We have previously shown that this insect has a range of color phenotypes—green, intermediate, or black, in response to the presence or absence of conspecifics (Silva et al. 2013). Here, we hypothesized that temperature affects the insect's movement rates, and thus indirectly affects the phenotype (visible first as color) of caterpillars reared in a group, that is, eight individuals per pot. While we did not measure movement (see above), we did find an effect of temperature on color phenotype, that was repeated in setting up the three experiments. In the three experiments, the frequency distributions of phenotypes of caterpillars reared in isolation were independent of temperature (encapsulation/melanization, *χ* ^*2*^_[6]_ = 4.4536, *P *=* *0.6155; hemocyte numbers, *χ* ^2^_[3]_ = 1.3009, *P *=* *0.7289; and infection by AgMNPV, *χ* ^*2*^_[6]_ = 4.3539, *P = *0.6289; Fig. 3A, C and E). In contrast, the frequency distributions of phenotypes were highly dependent on temperature when the caterpillars were kept in groups (encapsulation/melanization, *χ* ^*2*^_[6]_ = 17.1977, *P *=* *0.008; hemocyte count, *χ* ^2^_[6]_ = 39.5917, *P *<* *0.001; and infection by AgMNPV, *χ* ^*2*^_[6]_ = 20.9569, *P = *0.001). The differences are more specifically dependent on the temperature range to which caterpillars are submitted, that is, if they develop under extreme or mid‐range temperatures (contrast between extreme temperatures [20 and 32°C] and mid‐range temperatures [24 and 28°C]: encapsulation/melanization, *χ* ^*2*^_[2]_ = 15.3621, *P *<* *0.001; hemocyte count, *χ* ^2^_[2]_ = 38.6607, *P *<* *0.001; and infection by AgMNPV, *χ* ^*2*^_[2]_ = 16.4537, *P < *0.001; Fig. 3B, D and F). Group‐reared caterpillars exhibited significantly more of the black phenotype at the mid‐range temperatures, and less at the extreme temperatures. Meanwhile, the intermediate phenotype showed the opposite pattern; it was more frequently observed at the extreme temperatures, and less observed at the mid‐range temperatures. The expression of the green phenotypes remained pretty much constant with temperatures.

### Experiment 1: Effects of temperature and population density on immune defenses in caterpillars

#### Encapsulation response

**Figure 4:**
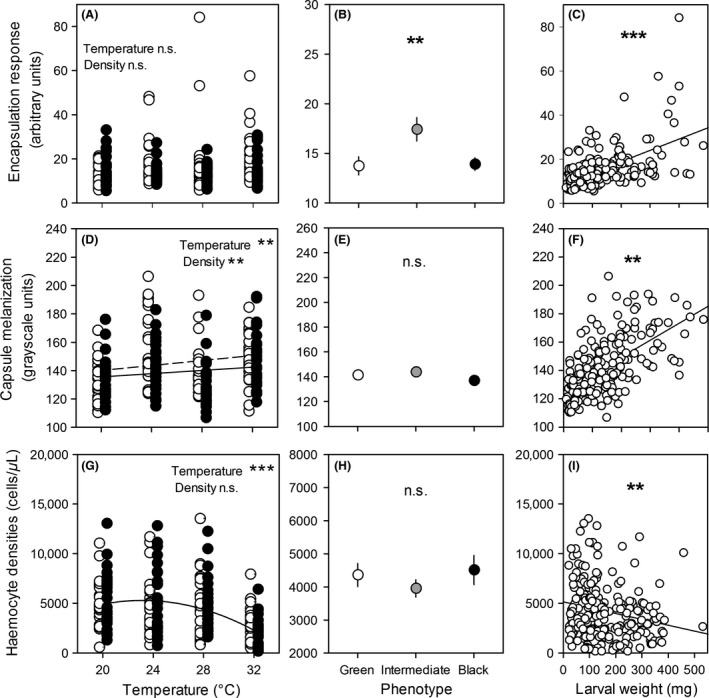
Effects of two environmental factors, temperature and population density as main factors (on the left panels), and larval phenotype and weight (on the central and right panels) used as covariates on immune defenses (encapsulation response: A–C, capsule melanization: D–F and hemocyte numbers: G–I) of velvetbean caterpillar. Caterpillars were kept alone (panels A, D, and G: open circles and dashed line) or in groups of 8 (panels A, D, and G: filled circles and solid line) in four thermostat‐controlled chambers with different temperatures (20, 24, 28, or 32°C) and weighed before the experiments as a measurement of body condition. The solid line in panel G applies to both densities as no significant difference was found between them. Error bars in panels B, E, and H are standard errors. Asterisks indicate significant *F*‐tests: **P < 0.01, ***P < 0.001; and n.s. nonsignificant terms.

The encapsulation response of the nylon filament was not affected directly by temperature (*P *=* *0.0538; Table 1; Fig. 4A) or population density (*P *=* *0.1194; Table 1; Fig. 4A). It did, however, vary with phenotype (*P *=* *0.0051; Table 1; Fig. 4B): caterpillars exhibiting the intermediate phenotype had a greater encapsulation response than green and black phenotypes (contrast analysis: *P *=* *0.0049; Table 1). It also increased with caterpillar weight (*P *<* *0.001; Table 1; Fig. 4C). No significant interaction was found between temperature and density (*P *=* *0.4095; Table 1).

**Table 1:** Analysis of deviance table testing the effects of environmental factors, (1) temperature (20, 24, 28, or 32°C), and (2) population density (1 or 8 larvae/pot) as main factors, and larval phenotype (green, intermediate or black) and weight used as covariates on immune defenses of velvetbean caterpillar: encapsulation response, capsule melanization, and hemocyte numbers

Immune defenses	Effect	df	Deviance	F	Pr(>F)
Encapsulation response	Temperature	1, 211	324.4	3.7588	0.0538
	Density	1, 210	211.0	2.4445	0.1194
	Temperature:density	1, 205	59.3	0.6829	0.4095
	Phenotype	2, 208	933.1	5.4054	**0.0051** **
	Weight	1, 207	4103.6	47.5422	**0.0001** ***
	*Phenotype (intermediate vs. green + black)*	1, 211	688.9	8.0765	**0.0049** **
Capsule melanization	Temperature	1, 211	2185.5	9.0278	**0.0029** **
	Density	1, 210	1904.3	7.8663	**0.0055** **
	Temperature:density	1, 205	228.9	0.9451	0.3321
	Phenotype	2, 208	1297.4	2.6796	0.0709
	Encapsulation response	1, 207	1150.4	4.7518	**0.0304** *
	Weight	1, 206	25465.3	105.1891	**0.0001** ***
Hemocyte numbers	Temperature	1, 228	227206361	34.2429	**0.0001** ***
	Density	1, 227	2253384	0.3396	0.5606
	Temperature:density	1, 222	19841938	3.0175	0.0837
	Phenotype	2, 225	12348703	0.9306	0.3958
	Temp^2^	1, 224	85409023	12.8722	**0.0004** ***
	Weight	1, 223	50643040	7.6326	**0.0062** **

Shown is the minimal adequate model containing all the main factors and covariates.

Contrasts (in italic) are model simplifications by excluding nonsignificant terms and/or grouping treatment factors which are not significantly different from each other.

Bold letters indicate significant *F*‐tests: *P <* *0.05, **P <* *0.01, ***P <* *0.001.

#### Capsule melanization

In this experiment, we could also assess the capsule melanization formed around the nylon filament. Here, temperature did directly affect capsule melanization (*P *=* *0.0029; Table 1; Fig. 4D) while caterpillars reared in isolation had greater capsule melanization than those reared in groups (*P *=* *0.0055). No significant interaction was found between temperature and density (*P *=* *0.3321; Table 1). Differently from the previous experiment, phenotype did not affect capsule melanization (*P *=* *0.0709; Table 1; Fig. 4E). Capsule melanization was also indirectly affected via changes in caterpillar body mass (*P *<* *0.001; Table 1; Fig. 4F). We included encapsulation response as a covariate in the capsule melanization model as this parameter may vary with encapsulation response; indeed, capsule melanization increased with encapsulation response (*P *=* *0.0304; Table 1).

### Experiment 2: Effects of temperature and population density on hemocyte numbers

Hemocyte numbers decreased with temperature (*P *<* *0.001; Table 1; Fig. 4G) but were unaffected by population density (*P *=* *0.5606; Fig. 4G). There was also no interaction between these factors (*P *=* *0.0837; Table 1; Fig. 4G). Hemocyte numbers did not change according to phenotype exhibited by caterpillars (*P *=* *0.3958; Table 1; Fig. 4H). Finally, hemocyte numbers decreased with caterpillars' weight (*P *=* *0.0062; Table 1; Fig. 4I).

### Experiment 3: Effects of temperature and population density on infection by *Anticarsia gemmatalis multiple nucleopolyhedrovirus* (AgMNPV)

**Figure 5:**
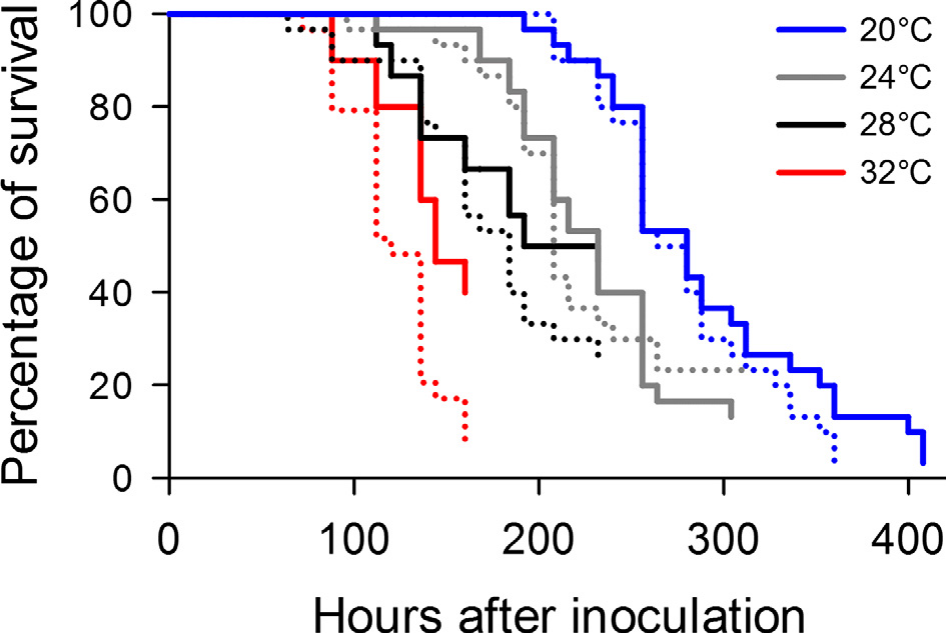
Survival curves of velvetbean caterpillar inoculated with AgMNPV. Caterpillars were kept alone or in groups of 8 in four thermostat‐controlled chambers with different temperatures. Dashed lines represent lone‐reared caterpillars; solid lines represent group‐reared caterpillars. Larval mortality was assessed daily, and the larvae that pupated were included in analysis as censored data. Survival analyses are presented in the text.

Survival times of velvetbean caterpillar infected by AgMNPV decreased with increasing temperature (*χ* ^2^_[5]_ = 258.62, *P *<* *0.001), but did not vary with population density (*χ* ^2^_[5]_ = 258.62, *P *=* *0.1824), and there was also no interaction between these two factors (*χ* ^2^_[5]_ = 258.62, *P *=* *0.9624; Fig. 5). The hazard ratio of velvetbean caterpillar decreased as temperature increased; this was seen during the analysis with the scale parameter lower than 1. The mean survival times of caterpillars reared at temperatures of 20, 24, 28, and 32°C were 282 ± 7 (±SE), 211 ± 7, 158 ± 7, and 123 ± 5 h, respectively. Although velvetbean caterpillars reared at 20 and 24°C lived longer after being infected, most succumbed at the end of the experiment (97 and 85% mortalities, respectively); while caterpillars reared at higher temperatures started dying earlier, the percentage mortality was lower (64% for 28°C and 78% for 32°C).

The caterpillars' survival was directly linked to the phenotype (*χ* ^2^_[5]_ = 258.62, *P *=* *0.001). While baculovirus‐infected caterpillars exhibiting green and intermediate phenotypes survived for mean times of 200 ± 10 (±SE) and 211 ± 9 h, respectively, those caterpillars exhibiting the black phenotype survived for mean times of 185 ± 9 h.

## Discussion

In the present study, temperature was found to affect two of the velvetbean caterpillars' biological parameters, larval body mass (which is related to food consumption) and immune responses. Environmental factors, not just temperature but population density as well, may also indirectly affect other biological parameters such as developmental time and contact between conspecifics. With changes in these parameters, immune responses may be up‐ or down‐regulated. Insects such as *Locusta migratoria* nymphs are able to adjust behaviorally their food and nutrient requirements according to a specific environmental temperature (Clissold et al. 2013), while some insects can balance the food consumption to achieve a better immunological response against pathogen attack (Povey et al. 2009). With regard to velvetbean caterpillar, some of its immune functions varied according to the environmental factors. The velvetbean caterpillar grows heavier at higher temperature, and this in turn increases the encapsulation response. Furthermore, caterpillars at both population densities presented greater capsule melanization at higher temperatures, although the most evident response was found in lone‐reared caterpillars. Although this finding is not in agreement with that predicted by the “density‐dependent prophylaxis” hypothesis (see Wilson and Reeson 1998), it matches previous results which show that lone‐reared caterpillars display more capsule melanization than group‐reared ones (Silva et al. 2013).

In contrast, temperature caused an opposite effect on hemocyte numbers; it affected negatively the hemocyte numbers in both lone‐ and group‐reared caterpillars at the same level. While some studies show this component of the immune function can be increased at warmer environmental temperatures (Truscott and White 1990; Chu and Lapeyre 1993), the decrease in hemocyte numbers found here may be related to its viability or stress‐induced damage (such as apoptosis) in a warmer environment (Yao and Somero 2012; Diaz et al. 2013). As hypothesized previously, immune parameters may change not only as a direct response to temperature, but they may change as an indirect response via changes in caterpillars' body mass. In this way, immune parameters correlated positively or negatively with velvetbean caterpillars' body mass. While encapsulation response and capsule melanization increased with body mass, hemocyte numbers decreased. Such correlations are also found in other species, for example, the tropical butterfly *Bicyclus anynana* in which hemocyte numbers correlate positively with thorax mass (Karl et al. 2011).

Beyond the immune parameters, temperature and population density also dramatically affect the host–pathogen system. Models suggest that warming increases baculovirus production and hence decreases time from inoculation to death of velvetbean caterpillar (known as viral developmental time) (Johnson et al. 1982; Ghosh and Bhattacharyya 2007). Our empirical data support this by showing that temperature, but not population density, affected considerably the developmental time; velvetbean caterpillar succumbed to AgMNPV more quickly at warmer temperatures (*ca*. 5 days) than at cooler ones (*ca*. 11 days). Nonetheless, the number of deaths (or mortality) was contrary to the warming (see Fig. 5). This may be a result of the host–pathogen interactions, as both host development time and pathogen virulence may be being affected by temperature changes.

Considering these results, it seems that the AgMNPV virulence is enhanced at higher temperatures. Thus, caterpillars die earlier as a function of the viral infection, but on the other hand, they develop faster as temperature increases (F.W.S.S. personal observation). In a closely related system, the fall armyworm *Spodoptera frugiperda* (Lepidoptera: Noctuidae), baculovirus transmission and epizootic progress increased with warming, while the insect's developmental time decreased (Elderd and Reilly 2014). In the system studied here, caterpillars that are not infected early by AgMNPV can escape infection by shifting from the larval stage to pupae, a mechanism known as “developmental resistance” (Hoover et al. 2002). For example, lepidopteran larvae become more resistant to baculovirus infection as they age (Engelhard and Volkman 1995; Kirkpatrick et al. 1998; Grove and Hoover 2007). We hypothesize that the mechanism behind the velvetbean caterpillars' resistance to AgMNPV is similar to that found in *Lymantria dispar*, that is, an increase in capsule melanization (McNeil et al. 2010). We thus expect the fitness of both pathogen and host to reach an “intermediate” level when environmental temperature is high. This is firstly because, as more hosts die from infection, more pathogen propagules can disperse to new hosts. However, those caterpillars that do not die from infection can reach adulthood and reproduce (Milks et al. 1998; El‐Sayed Hatem et al. 2011). On the other hand, pathogen fitness should be higher at lower temperatures as host mortality is maximum. Thus, most caterpillars die and the likelihood of viral dispersal increases. Meanwhile, host fitness will be pretty much zero as most of them die before reaching adulthood.

In a scenario of global warming, AgMNPV should evolve to maximize its virulence or speed of action at warmer environmental temperatures as its host can escape infection by shifting developmental stages. In contrast, the virus can evolve an opposite strategy at low temperatures as the host developmental resistance will take longer to occur. Low virulence may further evolve as a result of negative genetic correlations between speed of kill and production of infective viral particles, as the longer AgMNPV takes to kill its host, the more infective viral particles it can produce (see review in Cory and Myers 2003). Here, we propose that *A. gemmatalis* uses an “all‐or‐nothing” strategy; it invests more in development at high temperatures, increasing thus its chance of escaping from viral infection. However, if the strategy is different at low temperatures, that is, higher investment in immune defenses, it will fail, first because the up‐regulation of immune responses (in this case hemocyte numbers which is higher at low temperature) will not translate into an effective defense against AgMNPV. Furthermore, the investment in immunity may impair its development (Cotter et al. 2008; van der Most et al. 2011), thus caterpillars which do not develop faster may not benefit from developmental resistance and will succumb to viral infection.

Lastly, interactive effects between temperature and population density may create an emerging property in velvetbean caterpillar's morphology and physiology. Temperature may interfere directly with host movement rates (Ruf and Fiedler 2002; Cormont et al. 2011), which in turn can affect the likelihood of contact between conspecifics, and thus phenotypic changes in this species. Velvetbean caterpillar, as other polyphenic species, has evolved plastic prophylactic responses to cope with the increased risk of parasite infection at crowding (Barnes and Siva‐Jothy 2000; Wilson et al. 2002; Silva et al. 2013, 2016), with cuticle melanization being indicative of a prophylactic responses. So, here we consider this feature being a visible evidence of the velvetbean caterpillar's movement rates. Indeed, cuticle melanization is dependent on both temperature and population density. As hypothesized (see schematic framework Fig. 2), temperature likely affected caterpillars' movement rates, and thus the contact between conspecifics, which in turn affected the phenotypic expression of group‐reared caterpillars.

In all experiments, the frequency distribution of phenotypes of lone‐reared caterpillars was independent of temperature, while the frequency distribution of phenotypes is shown to be highly dependent on temperature when velvetbean caterpillar are kept in contact with conspecifics. The differences are more specifically dependent on the temperature range to which caterpillars are submitted, that is, whether they develop under extreme or median temperatures. Group‐reared caterpillars presented the black phenotype more frequently at intermediate temperatures and less frequently at extreme ones. This shows that extreme temperatures, whether low or high, may impair the caterpillars' movement, and thus the likelihood of contact between them. Despite some work showing temperature affecting directly the movement and distribution (Jian et al. 2004), and even the cuticular melanization of insects (Prokkola et al. 2013), here we take a different approach to examine how temperature may affect indirectly the morphological and physiological traits via changes in contact between conspecifics.

Our results suggest that environmental factors, mainly temperature, strongly affect both the course of disease in velvetbean caterpillar populations by changing the effectiveness of host immune functions against pathogens. Temperature affected differently the immune parameters assessed here, with no changes in encapsulation response, increasing capsule melanization and decreasing hemocyte numbers as temperature increases. It is likely that capsule melanization plays an important role as a defense mechanism of the velvetbean caterpillar against viruses, as the increase in capsule melanization at high temperatures matches up with the highest caterpillars' survival. As discussed above, this immune function may be an essential component of the velvetbean caterpillar's developmental resistance. It is also shown here how extreme temperatures may impact the population dynamics of this species, firstly by changing aspects of the host–pathogen interactions and second by changing the likelihood of contact between conspecifics. This last aspect may trigger plastic responses, and consequently the investment in immune functions toward pathogen attack. As an important soybean pest, velvetbean caterpillar may increase its damage on soybean fields under a scenario of global warming as caterpillars may achieve developmental resistance faster, and thus decrease their susceptibility to biological control by AgMNPV. This may also have implications for large‐scale production of AgMNPV, as it replicates *in vivo*. As the investment of caterpillars in antiparasite defenses increases with temperature and population density, it could be suggested that laboratory production uses solitary caterpillars at lower temperatures. Caterpillars at lower temperatures take longer to reach adulthood, thus more viral particles may be produced during this period.

## Conflict of Interest

None declared.
